# Withdrawal Note: NT-3 contributes to chemotherapy-induced neuropathic pain through TrkC-mediated CCL2 elevation in DRG neurons

**DOI:** 10.1038/s44319-025-00535-0

**Published:** 2025-11-26

**Authors:** Dilip Sharma, Xiaozhou Feng, Bing Wang, Bushra Yasin, Alex Bekker, Huijuan Hu, Yuan-Xiang Tao

**Affiliations:** 1 Department of Anesthesiology, New Jersey Medical School, Rutgers, The State University of New Jersey, Newark, NJ 07103 USA; 2 Department of Physiology, Pharmacology & Neuroscience, New Jersey Medical School, Rutgers, The State University of New Jersey, Newark, NJ 07103 USA; 3 Department of Cell Biology & Molecular Medicine, New Jersey Medical School, Rutgers, The State University of New Jersey, Newark, NJ 07103 USA

## Abstract

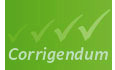

**Author withdrawal to:** *EMBO Rep* (2024) 25:2375-2390.

10.1038/s44319-024-00133-6| Published online 26 November 2025

This article has been withdrawn at the request of the authors following the identification of a mislabelling error in the manuscript. Specifically, proNT3 was incorrectly referred to as NT3 throughout the text.

Given the extent of the necessary textual revisions a corrected version of the paper under the title, “Proneurotrophin-3 in DRG neurons contributes to chemotherapy-induced neuropathic pain,” has been accepted for publication in *EMBO Reports*. This withdrawal is intended to avoid confusion between the original and corrected versions.

## Author statement

We recently discovered a labelling error in our manuscript titled “NT-3 contributes to chemotherapy-induced neuropathic pain through TrkC-mediated CCL2 elevation in DRG neurons” published in *EMBO Reports* (2024 May 25(5): 2375–2390 10.1038/s44319-024-00133-6).

Specifically, proNT3 was mistakenly referred to as NT3. As shown in the corrected version of the manuscript, NT3 is not detected in the dorsal root ganglia (DRG) of either naïve or paclitaxel-treated mice.

We have corrected this mislabelling and updated the manuscript accordingly (Sharma et al., 2025).

All authors agree to this withdrawal.
